# Exploration of Habitat-Related Chemical Markers for *Stephania tetrandra* Applying Multiple Chromatographic and Chemometric Analysis

**DOI:** 10.3390/molecules27217224

**Published:** 2022-10-25

**Authors:** Xiunan Cao, Xinxin Miao, Minglei Ge, Mengmeng Zhang, Zhenguo Lv, Wei Wang, Yanxu Chang, Huizi Ouyang, Jun He

**Affiliations:** 1 State Key Laboratory of Component-Based Chinese Medicine, Tianjin University of Traditional Chinese Medicine, Tianjin 301617, China; 2 Haihe Laboratory of Mordern Chinese Medicine, Tianjin 301617, China

**Keywords:** *Stephania tetrandra*, habitat-related chemical markers, UHPLC-Q-TOF-MS/MS, GC-MS, chemometric analysis

## Abstract

Geo-authentic herbs refer to medicinal materials produced in a specific region with superior quality. *Stephania tetrandra* S. Moore (*S. tetrandra*) is cultivated in many provinces of China, including Anhui, Zhejiang, Fujian, Jiangxi, Hunan, Guangxi, Guangdong, Hainan, and Taiwan, among which Jiangxi is the geo-authentic origin. To explore habitat-related chemical markers of herbal medicine, an integrated chromatographic technique including gas chromatography-mass spectrometry (GC-MS), ultra-high-performance liquid chromatography coupled with quadrupole time-of-flight mass spectrometry (UHPLC-Q-TOF-MS/MS) and ultra-high-performance liquid chromatography-mass spectrometry (UHPLC-MS/MS) combined with chemometric analysis was established. The established methods manifested that they were clearly divided into two groups according to non-authentic origins and geo-authentic origins, suggesting that the metabolites were closely related to their producing areas. A total of 70 volatile compounds and 50 non-volatile compounds were identified in *S. tetrandra*. Meanwhile, tetrandrine, fangchinoline, isocorydine, magnocurarine, magnoflorine, boldine, and higenamine as chemical markers were accurately quantified and suggested importance in grouping non-authentic origins and geo-authentic origins samples. The discriminatory analysis also indicated well prediction performance with an accuracy of 80%. The results showed that the multiple chromatographic and chemometric analysis technique could be used as an effective approach for discovering the chemical markers of herbal medicine to fulfill the evaluation of overall chemical consistency among samples from different producing areas.

## 1. Introduction

*S. tetrandra* is derived from the dried root of *Stephania tetrandra* S. Moore (*S. tetrandra*) [1], a perennial liana plant of the genus *Stephania*, belonging to the Menispermaceae family [2]. It was first recorded as medicine in Shen Nong’s Herbal Classic and suitable as a treatment for arthralgia associated with rheumatoid arthritis, wet beriberi, eczema, and inflamed sores, which acts as a diuretic, analgesic, and anti-inflammatory [2,3,4]. Currently, the compounds isolated and identified from *S. tetrandra* are mainly alkaloids which are critical for evaluating their therapeutic effects and quality [5]. However, only two characteristic components, tetrandrine and fangchinoline, were defined as the quantitative indexes recorded in the Chinese Pharmacopoeia 2020 edition [1]. Other major components with high content and extensive pharmacological properties, such as magnoflorine, magnocurarine, isocorydine, higenamine, and boldine, have been less studied so far [2]. Therefore, it was an urgent need to develop more potential chemical markers to better evaluate the holistic chemical features of *S. tetrandra* from different origins.

It is well accepted that medicinal herbs exert their efficacies through synergistic actions via “multi-components hitting multi-targets” of complex chemicals in the herbs [6,7], and integrating multiple methods simultaneously characterizing different kinds of components has been employed as a comprehensive strategy to evaluate the holistic quality, thus assure the efficacy of medicinal herbs [8]. Moreover, chemometrics provides a variety of good algorithms to explore and obtain more valuable chemical information [9,10,11,12,13,14,15]. Among these, discriminatory analysis is an effective tool for accurate prediction according to various characteristic values. The combination of multiple chromatographic techniques and chemometrics would provide a reliable method for the biomarker screening of herbal medicine.

In this study, an integrated chromatographic technique based on ultra-high-performance liquid chromatography coupled with quadrupole time-of-flight mass spectrometry (UHPLC-Q-TOF-MS/MS) and gas chromatography-mass spectrometry (GC-MS) was proposed to study the geo-herbalism of *S. tetrandra*. The habitat-related biochemical markers were further investigated using multiple pattern recognition models. Subsequently, seven main components were quantitatively compared using the validated ultra-high-performance liquid chromatography-mass spectrometry (UHPLC-MS/MS). A discriminant function equation was established to verify the accuracy of the chemical markers and achieve the prediction of the origin of *S. tetrandra*. The proposed strategy, which is comprehensive and effective, can be used to assist the application of *S. tetrandra* as well as related herbal medicine.

## 2. Results and Discussion

### 2.1. Method Validation

The relative standard deviations (RSDs) of retention time (Rt) and peak area for precision, repeatability, and stability were less than 1.0% and 7.7% using GC-MS and UHPLC-Q-TOF-MS/MS analysis (Supplementary Table S1), which validated that the established method was precise for differential component analysis of *S. tetrandra* from different origins.

The UHPLC-MS/MS method was verified in terms of linearity, lower limits of quantification (LLOQs), precision, repeatability, stability, and recovery. The results are shown in Supplementary Tables S2–S4, which revealed that the established method was precise enough for the simultaneous quantitative determination of seven compounds.

### 2.2. Identification of Volatile Components by GC-MS Analysis

GC-MS analysis was carried out to analyze the volatile components of *S. tetrandra* samples, and the relative contents of volatile compounds were calculated by peak area normalization. According to the database NIST08 and NIST08s, 70 volatile components, including alkanes, fatty acids, esters, carbonyls, alcohols, and phenols, were identified. Among them, the content of esters was the highest, accounting for 28.44%, of which methyl (9E)-9-octadecenoate, methyl linoleate, and methyl palmitate were 11.48%, 6.50%, and 4.50%, respectively. Alkanes have the most types, with a relative content of 14.36%, of which 2,4-dimethyl-1-heptene was the most abundant, reaching 1.80%. The relative content of fatty acids was 10.26%, among which oleic acid and palmitic acid were the highest, which were 4.54% and 2.94%, respectively. The identification results are shown in Table 1, and the corresponding peak of each compound was exhibited in the total ion chromatograms (TIC) diagrams (Supplementary Figure S1).

### 2.3. Identification of Nonvolatile Components by UHPLC-Q-TOF-MS/MS Analysis

Chromatographic data collected from UHPLC-Q-TOF-MS/MS were imported into Agilent Masshunter Qualitative Workstation Analysis B.07.00 (Agilent Technologies Inc., Santa Clara, CA, USA) for the identification of nonvolatile components of *S. tetrandra*. The identification of compounds is based on accurate mass, Rt, ion pattern, and MS/MS information. The obtained mass spectrograms were verified by: (a) matching with the instrument-generated molecular formula; (b) analyzing the structural information of metabolites acquired from the Metlin database (http://metlin.scripps.edu, accessed on 29 June 2022); (c) comparing with the fragment information of the standard samples; (d) combining with the compound information of the previous reports. According to the above-mentioned data acquisition and mining strategies, a total of 50 compounds, including 25 alkaloids, six amino acids, four amides, four fatty acids, two phenols, two purine derivatives, one phospholipid, one nucleoside, and five other compounds, were identified in *S. tetrandra*. The detailed information on the compounds is shown in Table 2, and the TIC figures of *S. tetrandra* under positive and negative ion modes are illustrated in Supplementary Figure S2.

### 2.4. Exploration of Habitat-Related Chemical Markers Based on Global Components

**Figure 1:**
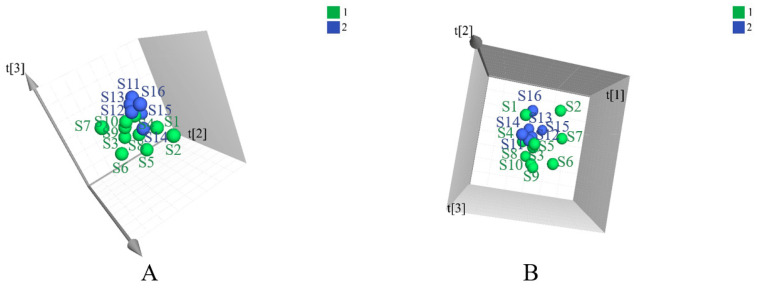
The PCA figures of two groups of *S. tetrandra* samples using UHPLC-Q-TOF-MS/MS analysis in the positive (**A**) and negative (**B**) ion models. (1: non-authentic origins; 2: geo-authentic origins).

**Figure 2:**
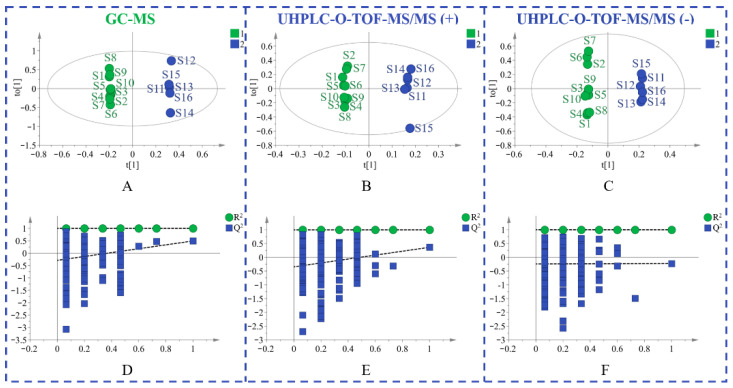
The OPLS-DA figures of two groups of *S. tetrandra* samples using GC-MS analysis (**A**), UHPLC-Q-TOF-MS/MS analysis in positive (**B**) and negative (**C**) ion model, validation of the model by a permutation test of GC-MS data (**D**), UHPLC-Q-TOF-MS/MS data in positive (**E**) and negative (**F**) ion model. (1: non-authentic origins; 2: geo-authentic origins).

Principal component analysis (PCA) is an unsupervised pattern recognition method that is often used to sort unclassified samples into groups. As displayed in Figure 1, samples from different provinces were separated into two groups. The geo-authentic samples had a more clustered distribution and homogeneous quality, whereas the non-authentic samples had a wide range of interval distribution and large differences in quality. Supervised orthogonal partial least squares discriminant analysis (OPLS-DA) was subsequently used to filter out random noises, distinguish differences between groups, and improve the validity and analytical ability of the model. The results in Figure 2A–C showed that the medicinal materials were grouped into two groups according to the non-authentic and geo-authentic origins. The model was conducted using 7-fold cross-validation in this research. R^2^ describes the goodness of fit and the cross-validation parameter, Q^2^, represents the predictive ability of the model. The constructed model had good quality, with a cumulative R^2^Y of 0.997 and Q^2^Y of 0.491. Permutation tests were conducted 200 times to assess whether the model was overfitted. As shown in Figure 2D–F, the blue regression lines of the Q^2^ points intersected the vertical axis below zero, and the intercepts of all regression lines on the vertical axis were less than 0.5; therefore, the model was reliable and not overfitted.

**Figure 3:**
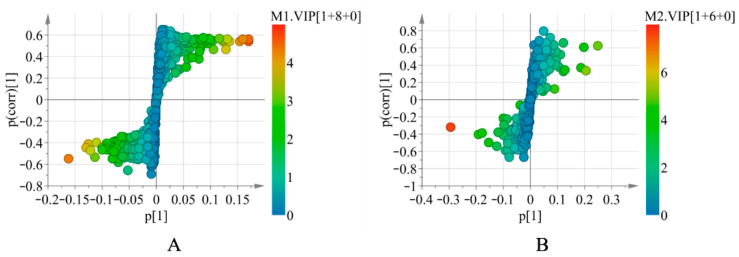
The OPLS-DA S-plots of *S. tetrandra* by GC-MS analysis (**A**) and UHPLC-Q-TOF-MS/MS analysis in positive ion mode (**B**).

In addition, based on the above models, the variables with the variable importance in the projection (VIP) > 1 were explicitly detected. To further visualize the components with VIP > 1, OPLS-DA was performed to generate S-plots (Figure 3). Finally, 14 differential volatile components (Supplementary Table S5) and 14 nonvolatile markers (Supplementary Table S6) were characterized, all of which played a significant role in the differentiation of different origins of *S. tetrandra*.

### 2.5. Quantitative Analysis of Habitat-Related Chemical Markers Based on Nonvolatile Components

Alkaloids are the main active ingredients and pharmacodynamic substances in *S. tetrandra*. Among them, tetrandrine, fangchinoline, isocorydine, magnocurarine, magnoflorine, boldine, and higenamine have many pharmacological activities, such as antimicrobial effects, anti-inflammatory, anticancer, immunomodulatory effects, and antiplatelet effects [2,12,13,14,15]. Thus, they were picked as habitat-related chemical markers for further quantitative analysis.

**Figure 4:**
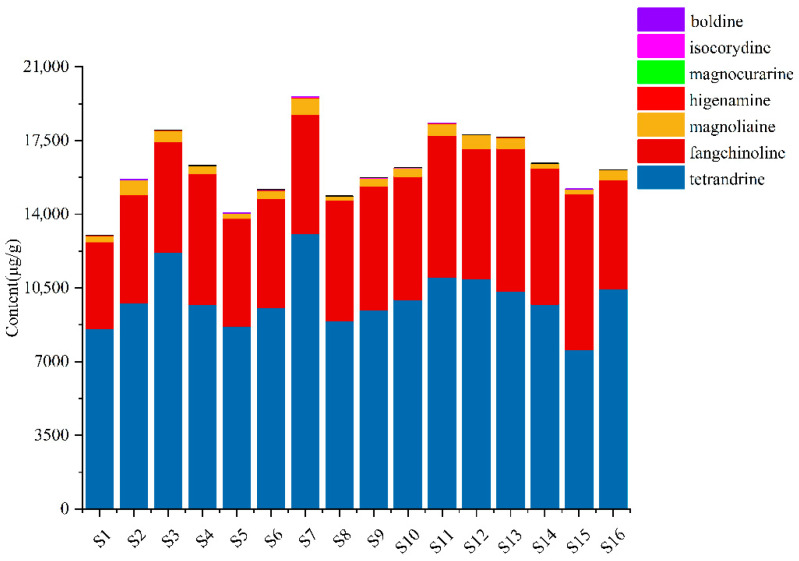
The total contents of seven alkaloids of *S. tetrandra* from different batches.

**Figure 5:**
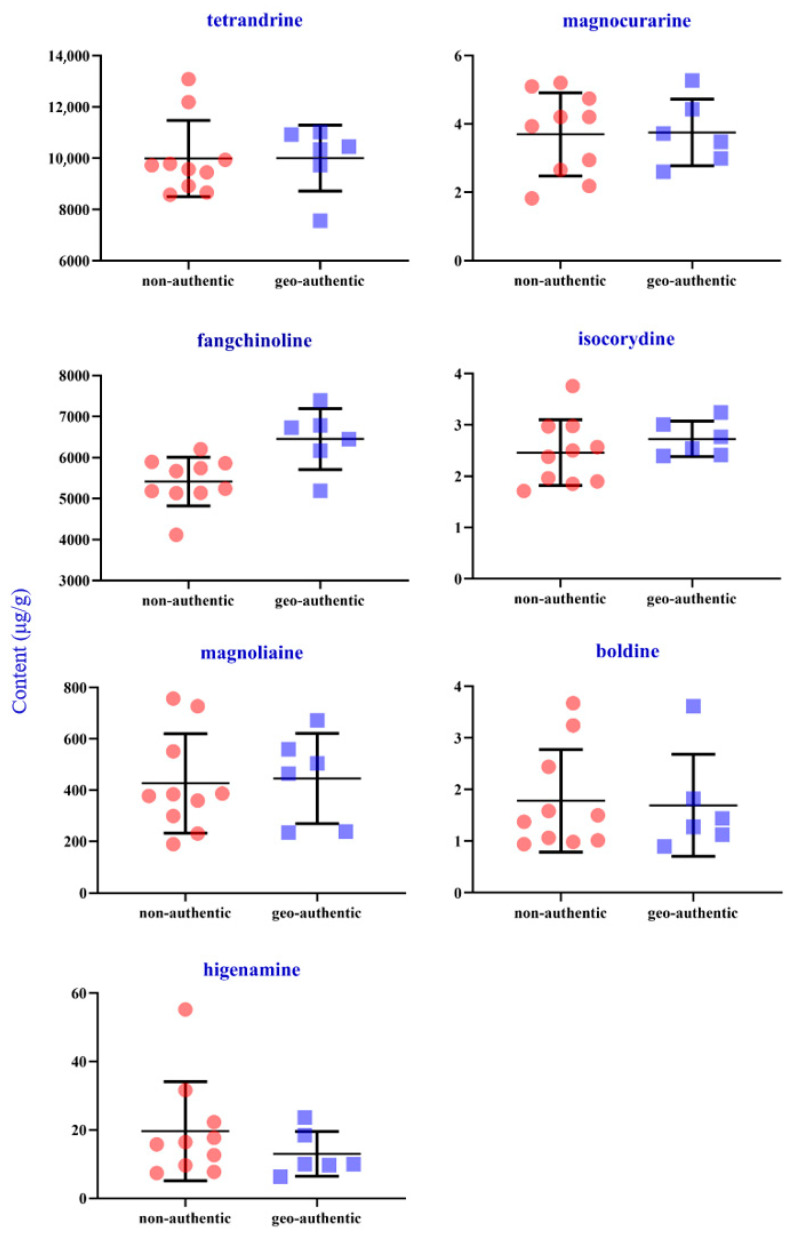
Contents of seven alkaloids of *S. tetrandra* from non-authentic origins and geo-authentic origins.

The structural formulas and detailed content of seven analytes in the 16 samples from different origins are exhibited in Figure S3 and Supplementary Table S7. The contents of seven compounds in sample extracts from different origins were inconsistent, which indicated that the different growth conditions, such as climate, and sunlight of different origins, may influence the quality of *S. tetrandra*. As shown in Figure 4, the total contents of analytes in samples from geo-authentic origins (S11–S16), especially the characteristic components, tetrandrine and fangchinoline, were higher. Besides, the contents of tetrandrine, fangchinoline, isocorydine, and higenamine in samples from geo-authentic origins were highly consistent (Figure 5). In contrast, the compound contents in non-authentic origin samples showed high fluctuation, and the contents of the S1 and S5 samples were low.

### 2.6. Discriminatory Analysis

Discriminant analysis is a multivariate statistical analysis method to determine the classification of research objects according to various characteristic values under the condition of a classification determination. In this study, establishing the domain U = {X1, X2, …, X11}, representing 11 randomly selected samples of *S. tetrandra*, and selecting the content of seven characteristic peaks such as tetrandrine, fangchinoline, magnoflorine, higenamine, magnocurarine, isocorydine, and boldine as the discriminant factors to form an 11 × 7 matrix. Then, by SPSS 21.0 (IBM, San Diego, CA, USA), Wilk’s lambda method was used for stepwise discriminant analysis, and a discriminant function for determining the origin of *S. tetrandra* was obtained. The regression estimation method was used to evaluate the superiority and inferiority of the discriminant function. Finally, the discriminant function equation of *S. tetrandra* was obtained as follows (S1: tetrandrine, S2: fangchinoline, S3: magnoflorine, S4: higenamine, S5: magnocurarine, S6: isocorydine, S7: boldine):

Y1 = 0.069S1 + 0.073S2 − 0.157S3 − 0.957S4 − 4.415S5 − 79.374S6 − 30.520S7 − 376.515 (non-authentic origins)

Y2 = 0.069S1 + 0.075S2 − 0.155S3 − 1.056S4 − 7.327S5 − 70.871S6 − 30.181S7 − 399.405 (geo-authentic origins)

By taking the content of each characteristic peak after screening into the function equation and comparing the Y values of the function equations from different origins, the value of which is the largest belonging to the origin represented by this equation. Through the test of the regression estimation method, we tested another five batches of *S. tetrandra* of known origin, the discriminant analysis of the source origin was compared with the actual results, and the correct rate was 80% (Table 3). This showed that the discriminant function equation established was relatively stable, can achieve the prediction and identification of the origin of *S. tetrandra*, and is valuable in promotion and application.

## 3. Materials and Methods

### 3.1. Chemical Reagents and Materials

Tetrandrine, fangchinoline, magnoflorine, magnocurarine, isocorydine, higenamine, and boldine were collected from Chengdu DeSiTe Biological Technology Co., Ltd. (Chengdu, China). Chromatographic grade methanol and acetonitrile were provided by Thermo Fisher Scientific Co., Ltd. (Shanghai, China). Formic acid was purchased from ROE Co., Ltd. (St. Louis, MO, USA). The Milli-Q system (Millipore, Bedford, MA, USA) was used to obtain purified water.

Sixteen batches of *S. tetrandra* were collected from seven different provinces (Guangxi, Guangdong, Sichuan, Neimeng, Anhui, Zhejiang, and Jiangxi) in China. The sample information is shown in Supplementary Table S8.

### 3.2. Preparation of Standard and Sample Solutions

Stock solutions of tetrandrine, fangchinoline, magnoflorine, magnocurarine, isocorydine, higenamine, and boldine were dissolved with methanol at a concentration of 10 mg/mL and serially diluted to plot the standard curves.

All dried samples were grounded, and the powder was passed through a 50-mesh sieve. Powdered samples (1 g) were ultrasonically extracted in 10 mL of n-hexane for 30 min. After cooling, the resulting mixture was centrifuged, and the supernatant filtered through 0.22 µm nylon membranes was collected for GC-MS analysis.

Pulverized samples (0.5 g) were sonicated in 20 mL of 70% methanol for 30 min. After centrifugation, the supernatants were filtered through a 0.22 µm nylon membrane to obtain the sample solutions for LC-MS/MS analysis.

Meanwhile, all equivalent volumes (100 μL) of sample solutions for GC-MS and LC-MS/MS analysis were respectively mixed as quality control (QC) samples. These sample solutions were all stored at 4 °C until analysis.

### 3.3. GC-MS Analysis

The volatile components were analyzed by a QP 2010 GC-MS (Shimadzu, Kyoto, Japan). Chromatographic separation was performed on a DB-5MS column (0.25 µm × 0.25 mm × 30 m). The temperature program was set as follows: 40 °C for 0 min, 40–190 °C at 10 °C/min, 190–210 °C at 3 °C/min, 210–215 °C at 1 °C/min, 215–240 °C at 6 °C/min.

Mass spectrometry was performed in electron impact (EI) mode and full scan mode at a mass-to-charge ratio (*m/z*) of 50–1000. The temperatures of the ion source and interface were 230 °C and 250 °C, respectively.

### 3.4. UHPLC-Q-TOF-MS/MS Analysis

The UHPLC-Q-TOF-MS/MS system is comprised of an Agilent 1290 UHPLC instrument (Agilent Technologies Inc., Palo Alto, CA, USA) and Agilent 6520 Q-TOF mass spectrometer (Agilent Corporation, Santa Clara, CA, USA). Chromatographic separation was performed on a Waters ACQUITY UPLC^®®^CSHTM C18 column (2.1 × 100 mm, 1.7 µm) at a temperature of 30 °C. The mobile phase consisted of 0.1% formic acid in water (solvent A) and acetonitrile (solvent B) at a flow rate of 0.2 mL/min, with a gradient elution program of 5–14% B at 0–5 min, 14–18% B at 5–12 min, 18–34% B at 12–14 min, 34–72% B at 14–22 min, 72–86% B at 22–23 min, 86–95% B at 23–31 min, and 95–95% B at 31–35 min. The injection volume for each sample was 5 μL.

The mass spectrometer was operated in both positive and negative modes with a scanning range of *m/z* 50–1500 and a scanning rate of 1 spectra/s. High resolution (4 GHz, High Res Mode) was used. The optimized instrumental parameters were as follows: capillary temperature, 350 °C; drying gas (N_2_) flow rate, 8 L/min; nebulizer pressure, 25 psi; collision energy, 30 V; fragment voltage, 135 V.

### 3.5. UHPLC-MS/MS Analysis

Quantitative analysis was performed on an Agilent 1290 UHPLC system (Agilent Technologies Inc., Palo Alto, CA, USA) equipped with an Agilent 6470 Triple quadrupole tandem mass spectrometer (Agilent Technologies, Singapore) with electrospray ionization (ESI) source. A Waters ACQUITY UPLC^®®^ BEH C18 column (2.1 × 100 mm, 1.7 µm) was used for chromatographic separation, and the column temperature was maintained at 20 °C. The binary gradient elution system consisted of 0.05% formic acid in water (A) and acetonitrile (B). The gradient profile started from 10% B, increased linearly to 23% B within 2 min, and then increased to 50% B within 3 min.

The mass spectrometer was operated in positive mode. The optimized mass conditions were as follows: gas temperature, 320 °C; gas flow rate, 8 L/min; nebulizer, 35 psi; sheath gas temperature, 250 °C; sheath gas flow, 12 L/min; capillary voltage, 4000 V. Multiple reaction monitoring (MRM) mode was applied for the quantitative analysis of different compounds. An MRM diagram is shown in Supplementary Figure S4. The optimal mass spectral parameters and ion patterns are presented in Table 4.

### 3.6. Method Validation

The GC-MS and UHPLC-Q-TOF-MS/MS methods were validated in terms of precision, repeatability, and stability. The precision was evaluated by observing the intraday variations of the QC sample six consecutive times. The repeatability was accessed by preparing six replicate QC samples. The stability was obtained by detecting one QC sample at 0, 6, 12, 18, and 24 h. Fifteen chromatographic peaks were randomly selected to calculate the RSDs of peak area and RT to investigate precision, repeatability, and stability.

The methodology of UHPLC-MS/MS analysis was verified by determining linearity, LLOQs, precision, repeatability, stability, and recovery. A calibration curve for each alkaloid standard was constructed using a linear regression model, and linearity was verified using correlation coefficients (r). The LLOQs were estimated as the minimum concentration giving signal-to-noise ratios (S/N) of 10. Instrument precision was determined by analyzing six replicates. Repeatability was evaluated by performing six replicate analyses on the same QC sample. In the stability test, the QC sample solutions were stored at room temperature and then analyzed by replicate injections at 0, 2, 4, 8, 12, and 24 h. The recoveries for spiked samples were applied to examine the effect of the extraction method and matrix effect. Blank *S. tetrandra* samples (0.25 g) and certain amounts of mixed standard solution were dissolved in 20 mL of 70% methanol and then processed by the optimized method, which were regarded as the spiked samples. The recovery rate was calculated using the following formula: recovery rate (%) = (observed amount − original amount)/spiked amount × 100%. Pulverized samples (0.5 g) were sonicated in 20 mL of 70% methanol for 30 min

### 3.7. Data Preprocessing

The GC-MS and UHPLC-Q-TOF-MS/MS data of *S. tetrandra* from different origins were exported in MZ format using the GC-MS Postrun (Shimadzu, Kyoto, Japan) and Agilent Masshunter Qualitative Analysis software packages, respectively. The peak finding, alignment, and filtering of the raw data were preprocessed using R 2.7.2 software (R Foundation for Statistical Computing, Vienna, Austria) to obtain the Rt, *m/z*, and peak strength of each compound. Finally, the obtained data were imported into Simca-P 14.1 (Umetrics, Umea, Sweden) for OPLS-DA. Potential chemical markers to differentiate the *S. tetrandra* from different origins were screened according to the VIP value. R^2^ and Q^2^ values were used to validate the model. R^2^ implied the explanation capability towards original data, and Q^2^ indicated the prediction ability of the model. The discriminant analysis function equation was established by SPSS 21.0 software.

## 4. Conclusions

In conclusion, by combing the volatile and nonvolatile components based on multiple chromatographic analyses, the habitat-related chemical markers of *S. tetrandra* were discovered. Through integrated chemometrics analysis, 14 volatile components and 14 non-volatile oils were screened out as the important contributors to the chemical difference between geo-authentic and non-authentic origins samples. Among these, tetrandrine, fangchinoline, isocorydine, magnocurarine, magnoflorine, boldine, and higenamine as chemical markers with abundant pharmacological activities were quantitatively analyzed by UHPLC-MS/MS. The results showed that the total content of analytes in samples from geo-authentic origins was higher and more consistent. Finally, discriminant analysis was used to simulate the function equation representing the origin of *S. tetrandra* to trace the origin of medicinal materials, and it also verified the accuracy of the differential components obtained by OPLS-DA. The proposed method could aid the exploration of habitat-related chemical markers for other herbal medicines.

## Figures and Tables

**Table 1:** Volatile chemicals of *S. tetrandra* by GC-MS analysis.

No.	Rt (min)	Compound	CAS	Molecular Formula	Molecular Weight	Retention Index	Similarity	Relative Content (%)
1	3.71	2,4-dimethyl-1-heptene	19549-87-2	C_9_H_18_	126	819	96	1.80
2	4.05	4-methyloctane	2216-34-4	C_9_H_20_	128	852	94	0.21
3	5.62	2-[(2-methylpropan-2-yl) oxy] oxolane	1927-59-9	C_8_H_16_O_2_	144	1024	82	0.07
4	6.12	butyl-2,2-dimethylpropanoate	5129-37-3	C_9_H_18_O_2_	158	999	87	0.07
5	6.26	3,3,5-trimethylheptane	7154-80-5	C_10_H_22_	142	867	94	0.67
6	6.33	3,3-dimethyloctane	4110-44-5	C_10_H_22_	142	931	94	0.75
7	6.90	5-(2-methylpropyl) nonane	62185-53-9	C_13_H_28_	184	1185	88	0.04
8	7.41	8-methylnonyl methacrylate	29964-84-9	C_14_H_26_O_2_	226	1483	88	1.08
9	7.79	4-methylundecane	2980-69-0	C_12_H_26_	170	1150	93	0.32
10	7.91	2,6,6-trimethyl-octane	54166-32-4	C_11_H_24_	156	966	93	0.31
11	8.18	(5-methyl-2-propan-2-ylhexyl) acetate	40853-55-2	C_12_H_24_O_2_	200	1189	82	0.05
12	8.54	nonadecane	629-92-5	C_19_H_40_	268	1910	81	0.02
13	9.23	dodecane	112-40-3	C_12_H_26_	170	1214	93	0.03
14	9.54	2,4-dimethylbenzaldehyde	15764-16-6	C_9_H_10_O	134	1208	97	0.28
15	10.31	hexadecane	544-76-3	C_16_H_34_	226	1612	92	0.29
16	10.68	11-methyldodecan-1-ol	27458-92-0	C_13_H_28_O	200	1492	89	1.30
17	10.80	2,4-diethylheptan-1-ol	80192-55-8	C_11_H_24_O	172	1229	88	1.73
18	10.92	2-hexyl-1-decanol	2425-77-6	C_16_H_34_O	242	1790	86	1.42
19	10.97	4-methyldodecane	6117-97-1	C_13_H_28_	184	1249	90	0.53
20	11.10	tetramethyl-2,3,6,7 octane	52670-34-5	C_12_H_26_	170	958	91	0.41
21	11.22	4,8-dimethylundecane	17301-33-6	C_13_H_28_	184	1185	90	0.58
22	11.55	3,3-dimethylhexane	563-16-6	C_8_H_18_	114	732	83	0.03
23	11.63	2,3-dimethyldecane	17312-44-6	C_12_H_26_	170	1086	88	0.05
24	12.04	tetradecane	629-59-4	C_14_H_30_	198	1413	95	0.06
25	12.77	2,3-dimethyldodecane	6117-98-2	C_14_H_30_	198	1285	90	0.33
26	12.88	2,6,10-trimethyldodecane	3891-98-3	C_15_H_32_	212	1320	88	0.56
27	13.12	4-methyltetradecane	25117-24-2	C_15_H_32_	212	1448	89	0.33
28	13.19	n-heptadecane	629-78-7	C_17_H_36_	240	1711	91	0.34
29	13.34	heptacosane	593-49-7	C_27_H_56_	380	2705	87	0.05
30	13.43	2,4-di-t-butylphenol	96-76-4	C_14_H_22_O	206	1555	95	1.51
31	13.66	3,7,11,15-tetramethylhexadecan-1-ol	645-72-7	C_20_H_42_O	298	1996	86	0.71
32	13.75	4,6,8-trimethylnon-1-ene	54410-98-9	C_12_H_24_	168	1012	89	1.33
33	14.00	pentatriacontane	630-07-9	C_35_H_72_	492	3500	87	0.96
34	14.14	n-heneicosane	629-94-7	C_21_H_44_	296	2109	91	0.31
35	14.21	octadecane	593-45-3	C_18_H_38_	254	1810	90	0.15
36	14.84	isopropyl dodecanoate	10233-13-3	C_15_H_30_O_2_	242	1615	90	0.13
37	15.35	phytane	638-36-8	C_20_H_42_	282	1753	87	0.14
38	15.69	4-ethylhexadecane	25117-26-4	C_17_H_36_	240	1647	88	0.17
39	15.76	8-methylheptadecane	13287-23-5	C_18_H_38_	254	1746	91	0.42
40	16.34	n-mocosane	629-97-0	C_22_H_46_	310	2208	89	0.71
41	16.47	nonahexacontanoic acid	40710-32-5	C_69_H_138_O_2_	998	7236	88	0.39
42	17.12	2-methyldodecane	1560-97-0	C_13_H_28_	184	1249	85	0.08
43	17.47	methyl pentadecanoate	7132-64-1	C_16_H_32_O_2_	256	1779	92	0.08
44	18.03	diisobutyl phthalate	84-69-5	C_16_H_22_O_4_	278	1908	81	0.05
45	19.11	methyl palmitate	112-39-0	C_17_H_34_O_2_	270	1878	95	4.50
46	19.41	tetradecyl ether	5412-98-6	C_28_H_58_O	410	2880	85	0.26
47	19.64	dotriacontane	544-85-4	C_32_H_66_	450	3202	87	0.48
48	19.75	palmitic acid	57-10-3	C_16_H_32_O_2_	256	1968	93	2.94
49	19.96	tetratetracontane	7098-22-8	C_44_H_90_	618	4395	89	0.52
50	20.12	tetrapentacontane	5856-66-6	C_54_H_110_	758	5389	87	0.36
51	20.22	hexatriacontane	630-06-8	C_36_H_74_	506	3600	81	0.22
52	22.35	methyl linoleate	112-63-0	C_19_H_34_O_2_	294	2093	96	6.50
53	22.51	methyl (9E)-9-octadecenoate	1937-62-8	C_19_H_36_O_2_	296	2085	94	11.48
54	22.62	methyl oleate	112-62-9	C_19_H_36_O_2_	296	2085	91	0.51
55	22.99	3-methylheptadecane	6418-44-6	C_18_H_38_	254	1746	85	0.31
56	22.99	squalane	111-01-3	C_30_H_62_	422	2619	84	0.31
57	23.11	methyl stearate	112-61-8	C_19_H_38_O_2_	298	2077	93	0.49
58	23.21	linoleic acid	60-33-3	C_18_H_32_O_2_	280	2183	92	1.51
59	23.39	oleic acid	112-80-1	C_18_H_34_O_2_	282	2175	93	4.54
60	23.92	alkynyl stearic acid	34450-18-5	C_18_H_32_O_2_	280	2165	81	0.88
61	24.07	ethyl oleate	111-62-6	C_20_H_38_O_2_	310	2185	84	1.47
62	24.64	hexacontane	7667-80-3	C_60_H_122_	842	5985	85	0.69
63	25.19	palmityl acetate	629-70-9	C_18_H_36_O_2_	284	1978	93	0.62
64	25.19	octacosyl acetate	18206-97-8	C_30_H_60_O_2_	452	3171	94	0.62
65	27.88	tetracosane	646-31-1	C_24_H_50_	338	2407	94	0.42
66	28.55	methyl icosanoate	1120-28-1	C_21_H_42_O_2_	326	2276	88	0.53
67	29.19	oleamide	301-02-0	C_18_H_35_NO	281	2228	83	0.20
68	29.62	nonacosane	630-03-5	C_29_H_60_	408	2904	85	0.33
69	30.22	2,2’-methylenebis (6-tert-butyl-4-methylphenol	119-47-1	C_23_H_32_O_2_	340	2788	93	3.20
70	30.42	stearyl acetate	822-23-1	C_20_H_40_O_2_	312	2177	84	0.26

**Table 2:** Possible non-volatile chemicals of *S. tetrandra* by UHPLC-Q-TOF-MS/MS analysis.

No.	Rt (min)	Loading Form	Molecular Formula	Precursor Ion	Fragment Ions	Difference (ppm)	Possible Compound	Structure Types	Reference
1	1.14	[M+H]^+^	C_6_H_14_N_4_O_2_	175.1182	158.0904, 116.0704	4.6	l-arginine	amino acids	[16]
2	1.20	[M+H]^+^	C_5_H_5_N_5_	136.0611	119.0356	5.1	adenine	purine derivatives	[17]
3	1.40	[M+H]^+^	C_5_H_9_NO_2_	116.0701	70.0659	4.3	proline	amino acids	[16]
4	1.43	[M+H]^+^	C_5_H_11_NO_2_	118.0861	58.0672	1.7	valine	amino acids	[16]
5	1.99	[M+H]^+^	C_6_H_13_NO_2_	132.1017	86.0970, 69.0716	1.5	2-amino-4-methylpentanoic acid	amino acids	[16]
6	2.01	[M+H]^+^	C_9_H_12_N_2_O_6_	245.0778	113.0339	−4.1	uridine	nucleoside	[17]
7	2.14	[M+H]^+^	C_5_H_5_N_5_O	152.0553	135.0292	9.2	guanine	purine derivatives	[17]
8	2.29	[M+H]^+^	C_5_H_7_NO_3_	130.0493	84.0447	4.6	l-pyroglutamic acid	amino acids	[16]
9	2.86	[M+H]^+^	C_9_H_11_NO_2_	166.0852	120.0805, 91.0576	6.6	phenylalanine	amino acids	[16]
10	3.34	[M]^+^	C_21_H_26_NO_4_	356.1847	280.1084	4.2	xanthoplanine	alkaloids	[18]
11	3.47	[M+H]^+^	C_16_H_17_NO_3_	272.1273	255.1017, 107.0441	2.9	higenamine	alkaloids	[19]
12	3.61	[M+H]^+^	C_23_H_29_NO_8_	448.1957	269.1175, 237.0916, 107.0438	2.0	*O*_6_-methylhigenamine,7-*O*-D-glucopyranoside	others	[18]
13	3.75	[M+H]^+^	C_24_H_31_NO_9_	478.2073	316.1557, 192.1000	−0.2	laudanosoline,6-mether,12-*O*-ß-D-glucopyranoside	others	[18]
14	4.60	[M+H]^+^	C_18_H_19_NO_4_	314.1382	297.0970, 282.1241, 265.0262, 177.0320	1.6	norisoboldine	alkaloids	[20]
15	4.68	[M+H]^+^	C_18_H_19_NO_3_	298.1425	269.1156, 254.0966	4.4	n-methylcrotsparine	alkaloids	[21]
16	5.23	[M+H]^+^	C_19_H_21_NO_4_	328.1541	151.0756, 178.0802	0.6	scoulerine	alkaloids	[22]
17	5.40	[M+H]^+^	C_17_H_19_NO_3_	286.1429	269.1167, 179.0821, 164.0664, 107.0448,	3.1	coclaurine	alkaloids	[21]
18	5.73	[M+H]^+^	C_19_H_23_NO_4_	330.1697	192.0893, 175.0211, 137.0146	0.9	reticuline	alkaloids	[23]
19	5.87	[M+H]^+^	C_19_H_21_NO_4_	328.1545	237.0906	−0.6	boldine	alkaloids	[23]
20	6.01	[M]^+^	C_20_H_24_NO_4_+	342.1693	297.1117, 282.0895, 265.0853	3.5	magnoflorine	alkaloids	[21]
21	6.42	[M+H]^+^	C_21_H_23_NO_5_	370.1642	207.0719, 190.0827, 188.0774	1.9	allocryptopine	alkaloids	[22]
22	6.55	[M+H]^+^	C_20_H_25_NO_4_	344.1842	207.0815, 192.1007	4.1	tembetarine	others	[18]
23	6.83	[M]^+^	C_19_H_24_NO_3_	314.1751	209.0974, 175.0708	1.6	oblongine	alkaloids	[18]
24	6.83	[M+H]^+^	C_19_H_23_NO_3_	314.1751	206.1106	0.1	armepavine	alkaloids	[18]
25	6.85	[M]^+^	C_19_H_24_NO_3_	314.1751	269.1163, 107.0463	1.6	magnocurarine	alkaloids	[24]
26	7.43	[M+H]^+^	C_22_H_27_NO_8_	434.1806	356.1492	0.7	fenfangjine G	alkaloids	[25]
27	7.56	[M+H]^+^	C_36_H_38_N_2_O_6_	595.2800	227.1051	0.5	2-northalrugosine	others	[18]
28	7.63	[M+H]^+^	C_20_H_23_NO_4_	342.1727	176.0717, 165.0911	−7.9	tetrahydrocolumbamine	alkaloids	[26]
29	7.70	[M+H]^+^	C_20_H_23_NO_4_	342.1717	311.1442	−5.0	isocorydine	alkaloids	[26]
30	8.58	[M+H]^+^	C_37_H_40_N_2_O_6_	609.2966	566.2543, 367.1639	−1.1	fangchinoline	alkaloids	[21]
31	9.57	[M+H]^+^	C_37_H_38_N_2_O_6_	607.2796	564.2370, 227.1038	1.2	cepharanthine	alkaloids	[21]
32	10.25	[M+H]^+^	C_21_H_25_NO_4_	356.1855	251.1071	0.3	glaucine	alkaloids	[17]
33	10.69	[M+H]^+^	C_38_H_42_N_2_O_6_	623.3118	580.2693	−0.3	tetrandrine	alkaloids	[18]
34	11.72	[M+H]^+^	C_20_H_21_NO_4_	340.1535	309.111	2.4	dicentrine	alkaloids	[26]
35	13.39	[M+H]^+^	C_18_H_19_NO_2_	282.147	191.0823, 207.0874	6.7	o-nornuciferine	alkaloids	[27]
36	15.32	[M+H]^+^	C_19_H_21_NO_2_	296.1641	235.0810, 191.0858	1.4	nuciferine	alkaloids	[27]
37	15.74	[M+H]^+^	C_19_H_19_NO_3_	310.1437	235.0759, 247.0758	0.3	fenfangjine F	alkaloids	[25]
38	16.12	[M+H]^+^	C_18_H_17_NO_2_	280.1319	249.0906, 219.0784, 191.0840	4.6	roemerine	alkaloids	[21]
39	16.34	[M+H]^+^	C_19_H_19_NO_2_	294.1482	233.0965	2.4	dehydeonuciferine	others	[27]
40	19.57	[M+H]^+^	C_18_H_39_NO_3_	318.2997	282.2769	1.9	phytosphingosine	phospholipid	[28]
41	25.99	[M+H]^+^	C_18_H_33_NO	280.2626	263.2352, 135.1150, 109.0908	3.2	linoleamide	amides	[29]
42	26.98	[M+H]^+^	C_18_H_30_O_2_	279.2306	261.2213	4.7	α-linolenic acid	fatty acids	[16]
43	27.51	[M+H]^+^	C_18_H_35_NO	282.2784	83.0867	2.5	oleamide	amides	[30]
44	28.45	[M+H]^+^	C_18_H_32_O_2_	281.2464	163.1380, 125.0847	3.9	linoleic acid	fatty acids	[30]
45	30.61	[M+H]^+^	C_18_H_34_O_2_	283.2616	171.1336, 265.2517	5.6	oleic acid	fatty acids	[31]
46	31.03	[M+H]^+^	C_16_H_33_NO	256.2624	102.0933	4.3	palmitamide	amides	[30]
47	34.51	[M+H]^+^	C_18_H_37_NO	284.2931	102.0924, 88.0722	6.0	stearamide	amides	[30]
48	11.78	[M−H]^−^	C_16_H_18_O_9_	353.0871	191.0633	−3.9	chlorogenic acid	phenols	[21]
49	13.73	[M−H]^−^	C_10_H_10_O_4_	193.0553	134.0312	2.4	ferulic acid	phenols	[32]
50	30.54	[M−H]^−^	C_18_H_36_O_2_	283.2665	197.0251	−7.8	stearic acid	fatty acids	[21]

**Table 3:** Effect evaluation of discriminant function equations of two groups of *S. tetrandra* samples.

Statistical Magnitude	Original Classification (Origin)	Discriminant Function Prediction Classification (Origin)		
		Non-Authentic Origins	Geo-Authentic Origins	Total
Number of samples	non-authentic origins	2	1	3
	geo-authentic origins	0	2	2
Percentage (%)	non-authentic origins	67	33	100
	geo-authentic origins	0	100	100

**Table 4:** Mass spectrometry parameters of seven target alkaloids.

Compound	Precursor Ion (*m/z*)	Product Ion (*m/z*)	Fragmentor (V)	Collision Energy (V)	Ion Mode
tetrandrine	623.2	580.1	80	45	Positive
fangchinoline	609.3	566.2	224	45	Positive
isocorydine	342.2	279.1	80	15	Positive
magnoflorine	342.1	297.1	80	30	Positive
higenamine	272.1	107.0	80	28	Positive
boldine	328.2	265.0	80	20	Positive
magnocurarine	314.2	269.1	113	20	Positive

## Data Availability

All datasets presented in this study are included in the article/Supplementary Material.

## Supplementary Materials

The following supporting information can be downloaded at: https://www.mdpi.com/article/10.3390/molecules27217224/s1, Figure S1: TIC diagram of *S. tetrandra* based on GC-MS analysis; Figure S2: TICs of *S. tetrandra* in positive (A) and negative (B) ions model using UHPLC-Q-TOF-MS/MS analysis; Figure S3: The structures of seven investigated alkaloids; Figure S4: MRM chromatograms of higenamine (1), magnocurarine (2), boldine (3), magnoflorine (4), isocorydine (5), fangchinoline (6), tetrandrine (7). (A) standard solution; (B) *S. tetrandra* sample; Table S1: The RSDs of precision, repeatability, and stability in GC-MS and UHPLC-Q-TOF-MS/MS analysis; Table S2: Linear equation, linear range, correlation coefficients (r) and lower LOQ of seven alkaloids in UHPLC-MS/MS analysis (n = 6); Table S3: RSDs of precision, repeatability, and stability of seven alkaloids in UHPLC-MS/MS analysis (n = 6); Table S4: The results of recovery test of seven alkaloids in UHPLC-MS/MS analysis (n = 6); Table S5: Potential volatile markers responsible for differentiation of *S. tetrandra* from non-authentic and geo-authentic origins; Table S6: Differential nonvolatile compounds responsible for differentiation of *S. tetrandra* from non-authentic and geo-authentic origins; Table S7: The contents of seven alkaloids in *S. tetrandra* from different origins (µg/g); Table S8: Source information of 16 batches *S. tetrandra* samples.

## Sample Availability

The *Stephania tetrandra* samples are available from the authors.
