# Supplementary material for: Exploration of Habitat-Related Chemical Markers for Stephania tetrandra Applying Multiple Chromatographic and Chemometric Analysis
Source: Molecules. 2022 Oct 25;27(21):7224. doi: 10.3390/molecules27217224 (PMC9654923; doi:10.3390/molecules27217224)
Supplement: Supplementary file 1 [file molecules-27-07224-s001.zip › molecules-1980483-supplementary.pdf]

Article

Exploration of habitat-related chemical markers for *Stephania tetrandra* applying multiple chromatographic and chemometric analysis

Xiunan Cao [1,2], Xinxin Miao [1], Minglei Ge [1], Mengmeng Zhang [1], Zhenguo Lv [1], Wei Wang [1], Yanxu Chang [1], Huizi Ouyang [1*] and Jun He [1,2*]

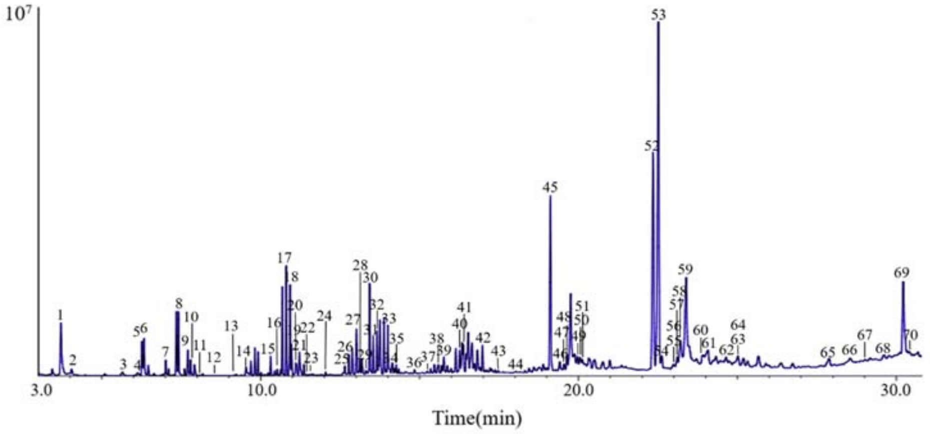

Figure S1. TIC diagram of *S. tetrandra* based on GC-MS analysis.

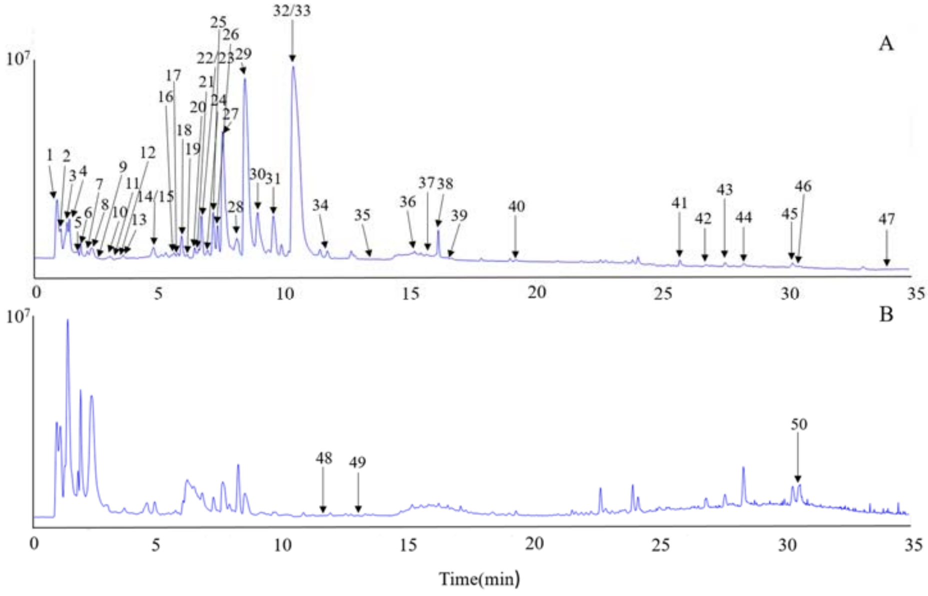

Figure S2. TICs of *S. tetrandra* in positive (A) and negative (B) ions model using UHPLC-Q-TOF-MS/MS analysis.

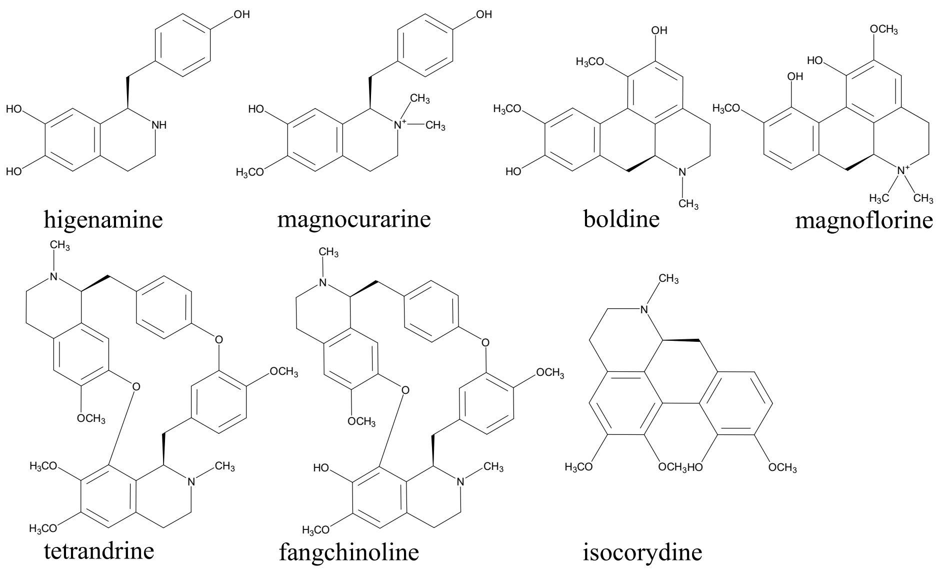

Figure S3. The structures of seven investigated alkaloids.

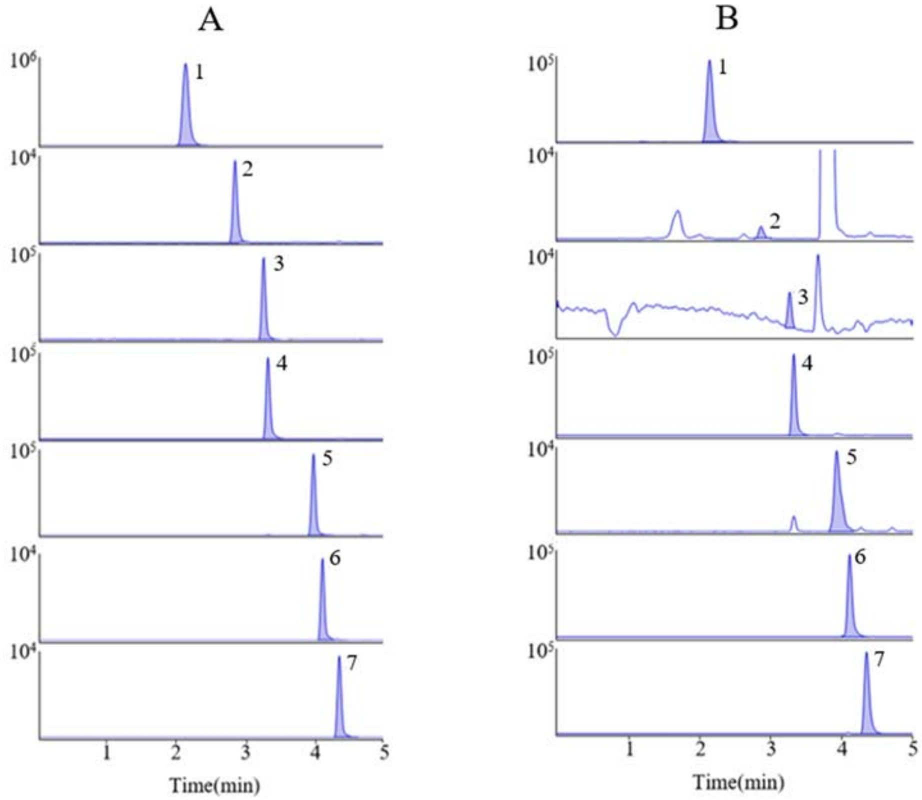

Figure S4. MRM chromatograms of higenamine (1), magnocurarine (2), boldine (3), magnoflorine (4), isocorydine (5), fangchinoline (6), tetrandrine (7). (A) standard solution; (B) *S. tetrandra* sample.

Table S1. The RSDs of precision, repeatability, and stability in GC-MS and UHPLC-Q-TOF-MS/MS analysis.

**Instrument**	**Type**	**Instrument Precision RSD (%)**	**Repeatability RSD (%)**	**Stability RSD (%)**
GC-MS/MS	Rt	< 0.1%	< 0.1%	< 0.1%
	Peak area	< 1.6%	< 7.4%	< 5.8%
UHPLC-Q-TOF-MS/MS	Rt	< 0.6%	< 0.1%	< 1.0%
	Peak area	< 5.9%	< 4.8%	< 7.7%

Table S2. Linear equation, linear range, correlation coefficients (r) and lower LOQ of seven alkaloids in UHPLC-MS/MS analysis (n = 6).

Compound	Linear Equation	r	Linearity Range (ng/mL)	LLOQ (ng/mL)
higenamine	y = 117131.6987x − 44149.1256	0.9997	1–500	0.05
magnocurarine	y = 5011.4155x − 1802.6231	0.9998	0.5–250	0.02
boldine	y = 2091402311x − 14145.6097	0.9998	1–500	1.00
magnoflorine	y = 1815.7697x − 4542.6748	0.9999	5–2500	0.20
isocorydine	y = 26805.9714x − 13439.0273	0.9998	1–500	0.03
fangchinoline	y = 96.1067x+5239.1087	0.9997	50–25000	0.03
tetrandrine	y = 69.8765x − 2792.4956	0.9997	100–50000	0.04

Table S3. RSDs of precision, repeatability, and stability of seven alkaloids in UHPLC-MS/MS analysis (n = 6).

**Compound**	**Instrument Precision RSD (%)**	**Repeatability RSD (%)**	**Stability RSD (%)**
higenamine	1.4	4.0	2.7
magnocurarine	2.7	3.3	2.3
boldine	3.8	2.9	3.7
magnoflorine	1.6	2.0	1.0
isocorydine	2.0	4.4	0.9
fangchinoline	1.1	1.1	1.0
tetrandrine	1.9	1.4	3.3

Table S4. The results of recovery test of seven alkaloids in UHPLC-MS/MS analysis (n = 6).

Compound	Observed amount (ng)	Original amount (ng)	Spiked amount (ng)	Average Recovery Rate (%)	RSD (%)
higenamine	10.5	5.6	5.0	98.5	4.9
magnocurarine	114.4	55.0	60.0	98.9	2.7
boldine	48.8	27.8	20.0	105.0	2.8
magnoflorine	8770.8	4358.0	4000.0	110.3	3.1
isocorydine	88.2	49.8	40.0	95.8	3.2
fangchinoline	237399.4	111587.6	120000.0	104.8	4.5
tetrandrine	385732.0	167991.2	200000.0	108.9	4.0

Table S5. Potential volatile markers responsible for differentiation of *S. tetrandra* from non-authentic and geo-authentic origins.

No.	Rt (min)	Compound	CAS	Molecular Formula	Molecular Weight	Retention Index	Similarity	Relative Content (%)
1	3.71	2,4-dimethyl-1-heptene	19549-87-2	C_9H_{18}	126	819	96	1.80
2	6.33	3,3-dimethyloctane	4110-44-5	$C_{10}H_{22}$	142	931	94	0.75
3	7.41	8-methylnonyl methacrylate	29964-84-9	$C_{14}H_{26}O_2$	226	1483	88	1.08
4	10.80	2,4-diethylheptan-1-ol	80192-55-8	$C_{11}H_{24}O$	172	1229	88	1.73
5	10.92	2-hexyl-1-decanol	2425-77-6	$C_{16}H_{34}O$	242	1790	86	1.42
6	13.43	2,4-di-t-butylphenol	96-76-4	$C_{14}H_{22}O$	206	1555	95	1.51
7	13.75	4,6,8-trimethylnon-1-ene	54410-98-9	$C_{12}H_{24}$	168	1012	89	1.33
8	19.11	methyl palmitate	112-39-0	$C_{17}H_{34}O_2$	270	1878	95	4.50
9	19.75	palmitic acid	21096	$C_{16}H_{32}O_2$	256	1968	93	2.94
10	22.35	methyl linoleate	112-63-0	$C_{19}H_{34}O_2$	294	2093	96	6.50
11	22.51	methyl (9E)-9-octadecenoate	1937-62-8	$C_{19}H_{36}O_2$	296	2085	94	11.48
12	23.11	methyl stearate	112-61-8	$C_{19}H_{38}O_2$	298	2077	93	0.49
13	23.39	oleic acid	112-80-1	$C_{18}H_{34}O_2$	282	2175	93	4.54
14	30.22	2,2'-methylenebis 6-tert-butyl-4-methylphenol	119-47-1	$C_{23}H_{32}O_2$	340	2788	93	3.20

Table S6. Differential nonvolatile compounds responsible for differentiation of *S. tetrandra* from non-authentic and geo-authentic origins.

No.	Rt (min)	Loading Form	Molecular Formula	Precursor Ion	Fragment Ion	Difference (ppm)	Possible Compound	Structure Types
1	1.14	$[M+H]^+$	$C_6H_{14}N_4O_2$	175.1182	158.0904, 116.0704	4.6	l-arginine	amino acids
2	2.29	$[M+H]^+$	$C_5H_7NO_3$	130.0493	84.0447	4.6	l-pyroglutamic acid	amino acids
3	3.47	$[M+H]^+$	$C_{16}H_{17}NO_3$	272.1273	255.1017, 107.0441	2.9	higenamine	alkaloids
4	5.40	$[M+H]^+$	$C_{17}H_{19}NO_3$	286.1429	269.1167, 179.0821, 164.0664, 107.0448,	3.1	coclaurine	alkaloids
5	5.73	$[M+H]^+$	$C_{19}H_{23}NO_4$	330.1697	192.0893, 175.0211, 137.0146	0.9	reticuline	alkaloids
6	5.87	$[M+H]^+$	$C_{19}H_{21}NO_4$	328.1545	237.0906	-0.6	boldine	alkaloids
7	6.01	$[M]^+$	$C_{20}H_{24}NO_4^+$	342.1693	297.1117, 282.0895, 265.0853	3.5	magnoflorine	alkaloids
8	6.85	$[M]^+$	$C_{19}H_{24}NO_3$	314.1751	269.1163, 107.0463	1.6	magnocurarine	alkaloids
9	7.43	$[M+H]^+$	$C_{22}H_{27}NO_8$	434.1806	356.1492	0.7	fenfangjine G	alkaloids
10	7.70	$[M+H]^+$	$C_{20}H_{23}NO_4$	342.1717	311.1442	-5.0	isocorydine	alkaloids
11	8.58	$[M+H]^+$	$C_{37}H_{40}N_2O_6$	609.2966	566.2543, 367.1639	-1.1	fangchinoline	alkaloids
12	10.25	$[M+H]^+$	$C_{21}H_{25}NO_4$	356.1855	251.1071	0.3	glaucine	alkaloids
13	10.69	$[M+H]^+$	$C_{38}H_{42}N_2O_6$	623.3118	580.2693	-0.3	tetrandrine	alkaloids
14	15.74	$[M+H]^+$	$C_{19}H_{19}NO_3$	310.1437	235.0759, 247.0758	0.3	fenfangjine F	alkaloids

Table S7. The contents of seven alkaloids in *S. tetrandra* from different origins (μg/g).

Sample	Tetrandrine	Fangchinoline	Magnoflorine	Higenamine	Magnocurarine	Isocorydine	Boldine
S1	8573.86	4114.77	299.21	7.72	1.82	1.85	0.98
S2	9780.96	5129.80	727.00	7.36	2.18	1.71	1.58
S3	12189.77	5237.57	551.30	12.61	4.74	3.76	3.24
S4	9720.53	6200.15	376.69	9.63	3.93	2.50	1.01
S5	8666.03	5137.59	230.44	22.29	2.94	1.90	1.06
S6	9566.78	5179.21	383.88	31.61	4.20	2.38	0.94
S7	13085.22	5669.37	757.08	55.23	5.20	2.98	3.67
S8	8919.54	5739.34	189.32	15.77	2.65	1.96	2.44
S9	9448.34	5894.30	358.82	16.45	5.10	2.57	1.37
S10	9937.63	5859.53	386.30	17.67	4.20	2.97	1.50
S11	11016.77	6725.79	559.98	18.44	5.27	3.24	3.61
S12	10927.58	6167.96	671.59	9.90	3.72	2.77	1.12
S13	10345.22	6779.81	504.45	23.60	4.43	3.01	1.82
S14	9725.53	6447.45	238.92	6.31	3.47	2.41	0.89
S15	7561.56	7392.52	234.51	9.92	2.60	2.54	1.27
S16	10448.61	5189.80	464.22	9.73	2.99	2.39	1.44

Table S8. Source information of 16 batches *S. tetrandra* samples.

Batch	Origin	Batch Number	Batch	Origin	Batch Number
S1	Yulin City, Guangxi Province	20191109	S9	Zhejiang Province	20190801
S2	Jieyang City, Guangdong Province	20191110	S10	Zhejiang Province	20191113
S3	Bozhou City, Anhui Province	20191107	S11	Wanzai County, Jiangxi Province	20191001
S4	Bozhou City, Anhui Province	20191201	S12	Jian City, Jiangxi Province	20191115
S5	Huoshan County, Anhui Province	20191111	S13	Jiujiang City, Jiangxi Province	20191008
S6	Guangyuan City, Sichuan Province	20191009	S14	Wuyuan County, Jiangxi Province	20191205
S7	Chifeng City, Neimeng	20190907	S15	Poyang Country, Jiangxi Province	20190301
S8	Zhejiang Province	20191106	S16	Fuzhou City, Jiangxi Province	20191105
